# Room-temperature Magnetism in Carbon Dots and Enhanced Ferromagnetism in Carbon Dots-Polyaniline Nanocomposite

**DOI:** 10.1038/s41598-017-01350-x

**Published:** 2017-05-19

**Authors:** Jian Liu, Hong Bi, Paulo Cesar Morais, Xiang Zhang, Fapei Zhang, Lin Hu

**Affiliations:** 1 College of Chemistry and Chemical Engineering, Anhui University, Hefei, 230601 China; 2 Department of Chemical and Chemical Engineering, Hefei Normal University, Hefei, 230601 China; 3 Universidade de Brasília, Instituto de Física, Brasília, DF 70910-900 Brazil; 4 High Magnetic Field Laboratory of the Chinese Academy Sciences, Hefei, 230031 China

## Abstract

Room temperature magnetic ordering is reported for very small carbon dots (CDs), mat-like polyaniline nanofibers (Mat-PANI) and a composite of CDs@Mat-PANI containing 0.315 wt% CDs. We have found saturation magnetization (*M*_*S*_) of CDs, Mat-PANI and CDs@Mat-PANI at 5 (20/300) K equals to 0.0079 (0.0048/0.0019), 0.0116 (0.0065/0.0055) and 0.0349 (0.0085/0.0077) emu/g, respectively. The *M*_*S*_ enhancement in CDs@Mat-PANI (200% and 40% at 5 K and 300 K, respectively) is attributed to electron transfer from Mat-PANI imine N-atoms to the encapsulated CDs. Changes in *M*_*S*_ values reveal that 0.81 (0.08) electron/CD is transferred at 5 (300) K, which is supported by observation of CDs photoluminescence (PL) redshift while in CDs@Mat-PANI. Band-bending and bandgap-renormalization calculations are used to predict a redshift of 117 meV at 300 K as a result of the electron transfer, in excellent agreement with the PL data (110 meV). Raman, X-ray diffraction and X-ray photoelectron spectroscopy data are used to confirm the electron transfer process as well as the strong interaction of CDs with PANI within CDs@Mat-PANI, which increases the crystalline domain size of Mat-PANI from about 4.8 nm to 9.2 nm while reducing the tensile strain from about 6.2% to 1.8%.

## Introduction

**Figure 1:**
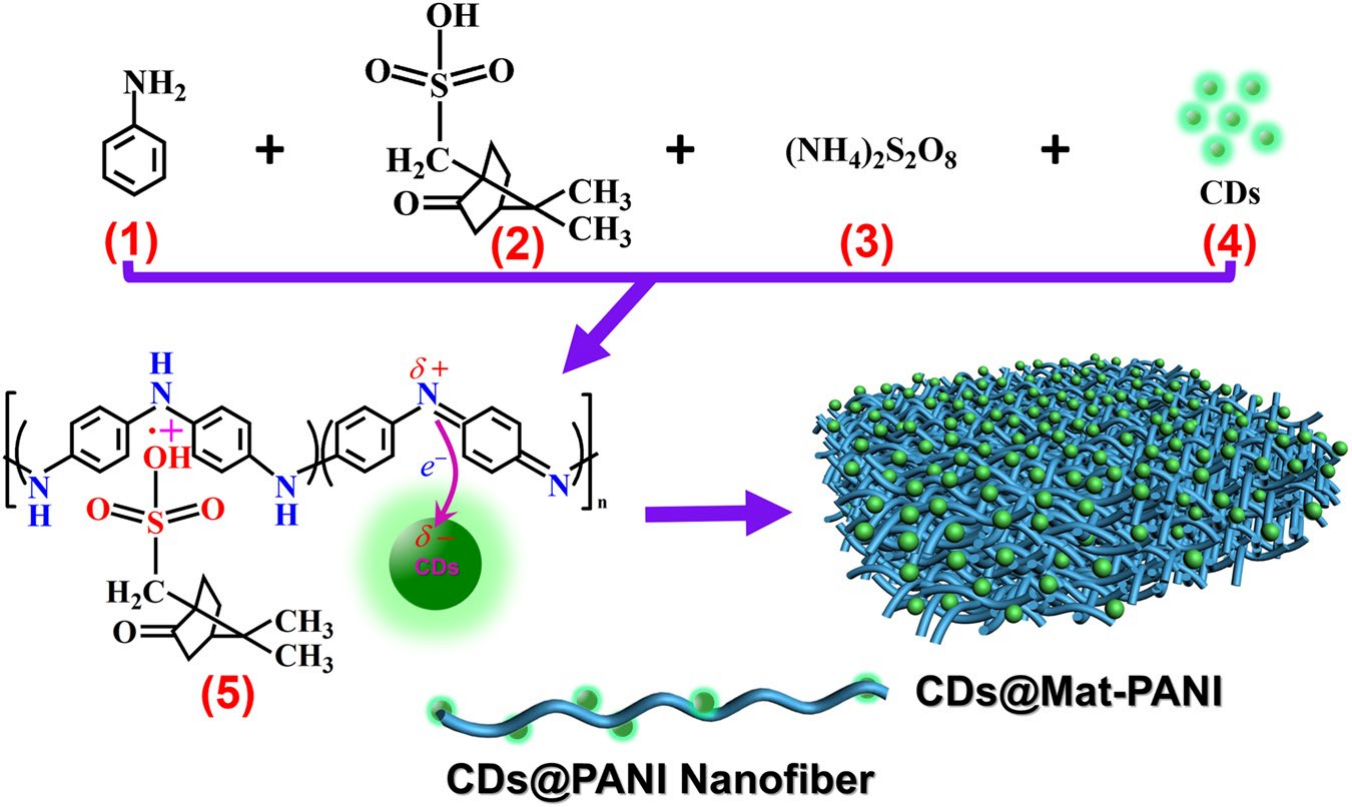
An illustration of the synthesis procedure of CDs@Mat-PANI.

The recent research and technology for new magnetic materials aim to combine room temperature operation, lightweight, low production cost, molding capability and environmental friendliness^1, 2^. Along this line metal-free carbon nanomaterials represent a very promising direction, as for instance in spintronics once carbon may easily integrate spin and molecular electronics into a single platform^3–5^. Room-temperature (RT) ferromagnetism in carbon nanomaterials was firstly reported in C_60_ while synthesized under high-pressure and high-temperature polymerization process^6, 7^. Subsequently, ferromagnetism in different carbon nanomaterials have been reported, e.g. diamonds up to 90 K^8^, carbon nanotubes up to 600 K^9^, and graphene quantum dots (GQDs) at 2 K^10, 11^. As a new member in carbon family, carbon dots (CDs) refer to nanoparticles with size below 10 nm that usually consist of a crystalline core with major sp^2^-hybridized carbon atoms plus an amorphous shell dominated by different chemical moieties, whose composition ultimately depends upon the carbon source used in the synthesis process^12, 13^. Recently, CDs were extensively explored in regard to their strong photoluminescence (PL) with high quantum yield as well as tunable surface defects and thus optical properties^14–17^. In the present study, we pioneering report on RT magnetic ordering in CDs, but also a new metal-free magnetic nanohybrid based on CDs encapsulated within polyaniline (PANI) nanofibers that have been assembled into a three-dimensional (3D) mat-like morphology (Mat-PANI). While RT ferromagnetism was already reported in PANI-based materials^18, 19^, enhancement of ferromagnetism promoted by integrating CDs and Mat-PANI into a single nanohybrid platform labeled as CDs@Mat-PANI is a novelty. In preparation of metal-free samples, much attention was given to the purity of initial reactants and careful synthesis and subsequent procedures in order to prevent samples from being contaminated by metal-based magnetic impurities. This is particularly important while examining weak RT magnetic ordering and its enhancement, as firstly reported here respectively in CDs and CDs@Mat-PANI, and yet not completely understood. Prior to the synthesis of the nanohybrid, CDs were prepared by pyrolysis of konjac flour (KF, Biosharp Company) and purified repeatedly using ethanol and distilled water according to the protocol described in our previous report^20^. Elemental analysis of the CDs indicated metal (Fe, Co, Ni, and Mn) trace level below 1 ppm. Figure 1 illustrates the synthesis procedure of CDs@Mat-PANI nanofibers and their entangled 3D mat-like structure. Although PANI with different morphologies had been extensively reported in the literature^21, 22^, mat-like morphology of PANI nanofibers is firstly reported here. We envisaged that the new family of magnetically-ordered CDs and CDs@Mat-PANI will offer multiple opportunities for fundamental studies, due to the modulation of their properties by varying the CDs characteristics, the PANI morphology and their relative content within the nanohybrid, opening up a new avenue for spintronics device fabrication.

## Results and Discussion

**Figure 2:**
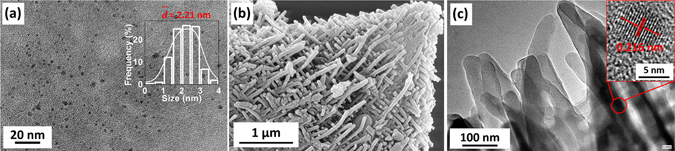
(**a**) TEM image of CDs (the inset is the particle size histogram of CDs); (**b**) SEM image of CDs@Mat-PANI; (**c**) HRTEM image of the brim nanofibers of CDs@Mat-PANI (the inset is a magnified image of an individual CD encapsulated within Mat-PANI nanofiber).

Figure 2a shows a typical transmission electron microscopy (TEM) image of the quasi-spherical CDs with an average diameter of 2.21 nm. The solid white-line going through the histogram in the inset of Fig. 2a is the best fitting of the data using a lognormal distribution function. Additionally, the inset of Fig. S1a in the Supplementary information shows 0.216 nm lattice fringe of an individual CD, indicating the existence of graphitic crystalline core in the CDs^12, 20^. Figure S1b shows UV-Vis absorbance spectrum of the CDs dispersed in ethanol with an obvious PL emission centred at 331 nm (*λ*_em_ = 310 nm). Figure 2b shows a representative scanning electron microscopy (SEM) image of the CDs@Mat-PANI, in which the above-described 3D mat-like morphology with thickness of 2–3 µm is clearly demonstrated. The corresponding magnified high-resolution TEM (HRTEM) image, as shown in Fig. 2c, confirms the presence of CDs encapsulated within the Mat-PANI nanofibers. The inset of Fig. 2c shows an individual CD in the PANI nanofiber with lattice diffraction fringes of 0.216 nm, which is in excellent agreement with that observed in pure CDs samples (see Fig. S1a). The control sample Mat-PANI shows a similar 3D mat-like morphology (see Fig. S2a,b), but without CDs in the PANI nanofibers (see Fig. S2c), indicating that addition of 0.315 wt% of CDs would not change the mat-like PANI morphology.

**Figure 3:**
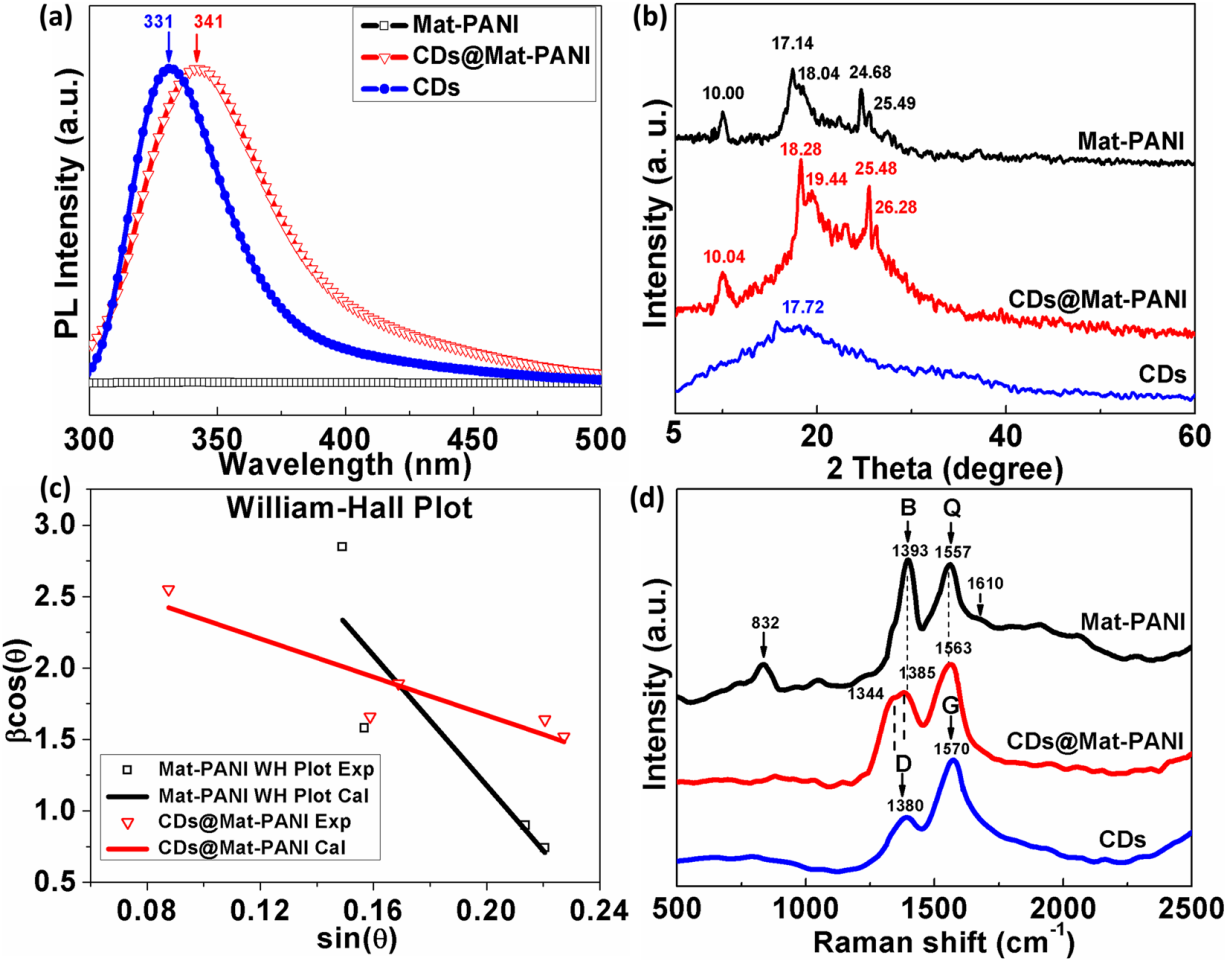
(**a**) PL spectra, (**b**) XRD patterns, (**c**) William-Hall plots of Mat-PANI and CDs@Mat-PANI, and (**d**) Raman spectra of Mat-PANI, CDs@Mat-PANI, and CDs.

Notably, Fig. 1 also illustrates electron transfer from PANI to CDs (see panel 5), which is assumed to occur and plays an important role in defining the very properties of the CDs@Mat-PANI. Figure 3a supports the electron transfer picture from Mat-PANI to CDs, for the RT redshift (from 331 nm to 341 nm) in the PL peak of sample CDs@Mat-PANI is credited to the enhancement of electron density held by the CDs, accompanied with the decrease of electron density in PANI backbone. Figure 3b shows the X-ray diffraction (XRD) data of the as-synthesized samples, where CDs reveal a broad XRD band with a small peak centred at 2*θ* = 17.72°, presenting d-spacing value of 0.413 nm, in good agreement with (002) plane of graphite^12^. The Mat-PANI sample exhibits three characteristic XRD peaks of crystalline CSA-doped PANI at 2*θ* = 10.00°, 18.04° and 24.68°, corresponding to d-spacing values of 0.888 nm, 0.497 nm and 0.369 nm for (010), (110) and (200) planes of protonic acid doped PANI, respectively^23, 24^. Besides the three crystalline-PANI-ascribed XRD lines, two other features (2*θ*) associated to the CDs@Mat-PANI and appearing in Fig. 3b should be explored as well (17.14° and 25.49°). The nanohybrid CDs@Mat-PANI presents clearer and sharper XRD peaks, indicating that its crystallinity has been improved in comparison with Mat-PANI. Analysis of the diffraction peaks in Fig. 3b reveals upshift of the Mat-PANI XRD lines after encapsulation of the CDs, suggesting tensile strain reduction in the nanohybrid^25^. Assessing five XRD features instead of three planes ((010), (110), and (200)), it is possible to draw the Williamson-Hall plot (*β*cos *θ* versus sin*θ*) using the Debye-Scherrer relation corrected for strain, i.e. *β*cos*θ* = (*kλ*/ < *D* > ) + 4*ε*sin *θ*, where *β*, *θ*, *k*, *λ*, < *D* > and *ε* are the corrected XRD linewidth, XRD angle, shape constant, XR wavelength, average grain size and strain, respectively^26^. Needless to mention the shape constant (*k*) must account for the high aspect ratio value of wire-like morphology of Mat-PANI nanofibers. From the W-H plots shown in Fig. 3c, one can assess the average crystallite grain size (< *D* >) and strain (*ε*) equal to 4.8 nm (9.2 nm) and 6.2% (1.8%) for Mat-PANI (CDs@Mat-PANI), respectively. Symbols in Fig. 3c are experimental data (W-H Plot Exp.) whereas solid lines represent the best fittings (W-H Plot Cal.) using the Debye-Scherrer equation mentioned in this paragraph. The *ε* value (1.8%) confirms reduction of tensile strain in the nanohybrid while increasing the crystallite average grain size by about 90% with respect to the Mat-PANI sample. This finding will be later on correlated with ferromagnetism enhancement observed in the nanohybrid.

Figure 3d shows two strong Raman features around 1380 and 1570 cm^−1^ in the CDs spectrum, accounting for sp^3^-hybridized (D) and sp^2^-hybridized (G) carbon-carbon vibrations, respectively^12^. Differently, Mat-PANI shows a Raman feature around 1557 cm^−1^, attributed to the C = N stretching vibration in quinomoid ring (Q)^27, 28^ whereas Raman features around 1393 and 1610 cm^−1^ had been ascribed to C-N^+^ vibration of delocalized polaronic structures (protonation band PB and B)^29, 30^ and C-C stretching of benzenoid ring^31, 32^, respectively. Raman bands appearing in the range of 700–900 cm^−1^ in the Mat-PANI spectrum (particularly 832 cm^−1^ in Fig. 3d) had been attributed to benzene ring or amine deformation in polaronic or bipolaronic forms of PANI^27^. When comparing Mat-PANI with CDs@Mat-PANI, the Raman spectrum of the latter reveals increased Q-band (1563 cm^−1^) intensity while reducing B-band (1385 cm^−1^) intensity, thus supporting the picture of electron transfer from imine Nitrogen atoms of PANI to CDs and then resulting in the decrease of electron density in PANI backbone. Compared with Mat-PANI, the CDs@Mat-PANI sample presents a shift of the C-N^+^ stretching peak towards much lower wavenumbers of 1385 cm^−1^ and 1344 cm^−1^ due to CDs encapsulation into PANI. Previously, CDs have been reported to exhibit unique photo-induced electron transfer as well as electron acceptor properties^33^. Interestingly, the Raman peak at 832 cm^−1^ is completely quenched in the nanohybrid (CDs@Mat-PANI), indicating change in the PANI backbone structure, more likely due to reduction of tensile strain as supported by the XRD peak upshift previously mentioned.

**Figure 4:**
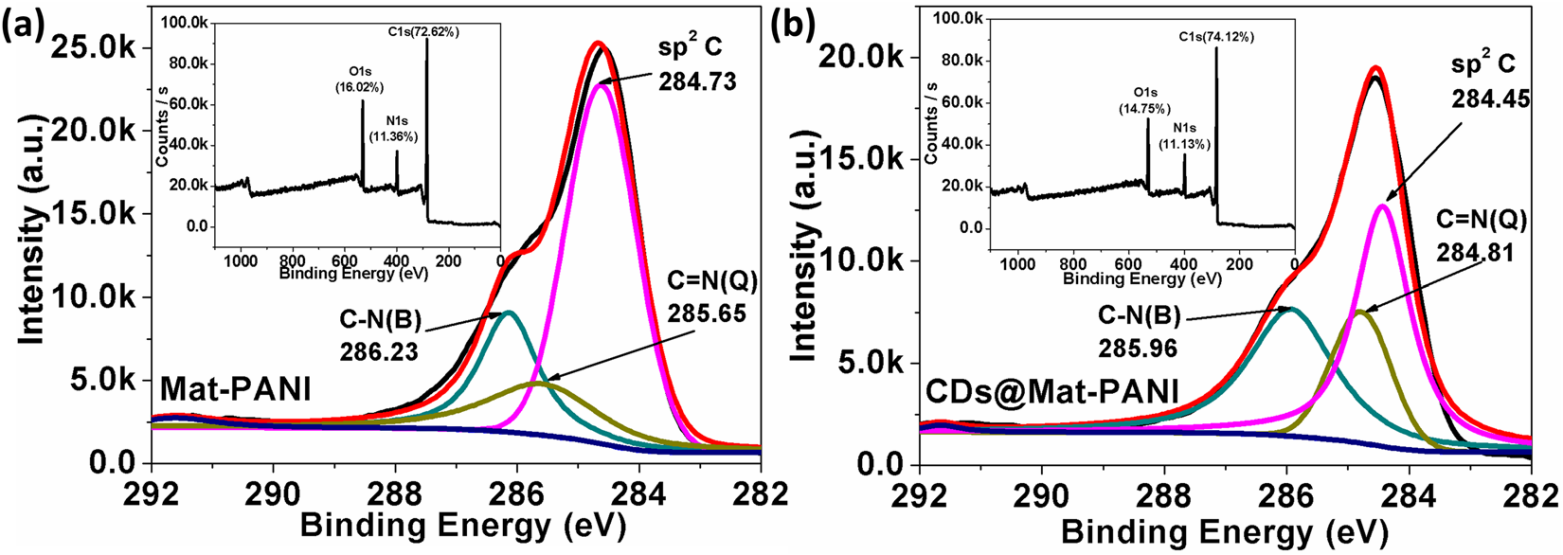
XPS C1s core-level spectra of (**a**) Mat-PANI and (**b**) CDs@Mat-PANI. The insets show general XPS spectra of Mat-PANI and CDs@Mat-PANI.

Further, Fig. 4a,b show the X-ray photoelectron spectroscopy (XPS) C1s core-level spectra of Mat-PANI and CDs@Mat-PANI, supporting the electron transfer process from Mat-PANI to CDs. The Gaussian fitted C1s binding energies of Mat-PANI at 284.73, 285.65 and 286.23 eV (see Fig. 4a) can be attributed to sp^2^C of benzenoid ring, C=N stretching vibration in quinomoid structure (labeled as C=N(Q)) and C-N^+^ delocalized polaronic structures (labeled as C-N(B)), respectively^34, 35^. Similarly, the C1s core level spectrum of CDs@Mat-PANI can be resolved into three Gaussian peaks at 284.45, 284.81 and 285.96 eV (see Fig. 4b), corresponding to sp^2^C, C=N(Q) and C-N(B) species, respectively. Note that the binding energies of sp^2^C, C=N and C-N^+^ species in CDs@Mat-PANI are all downshifted with respect to Mat-PANI, which can be ascribed to electron transfer from PANI to CDs in the nanohybrid, in good agreement with the Raman data shown in Fig. 3d. The insets of Fig. 4a,b show the general XPS spectra of Mat-PANI and CDs@Mat-PANI.

**Figure 5:**
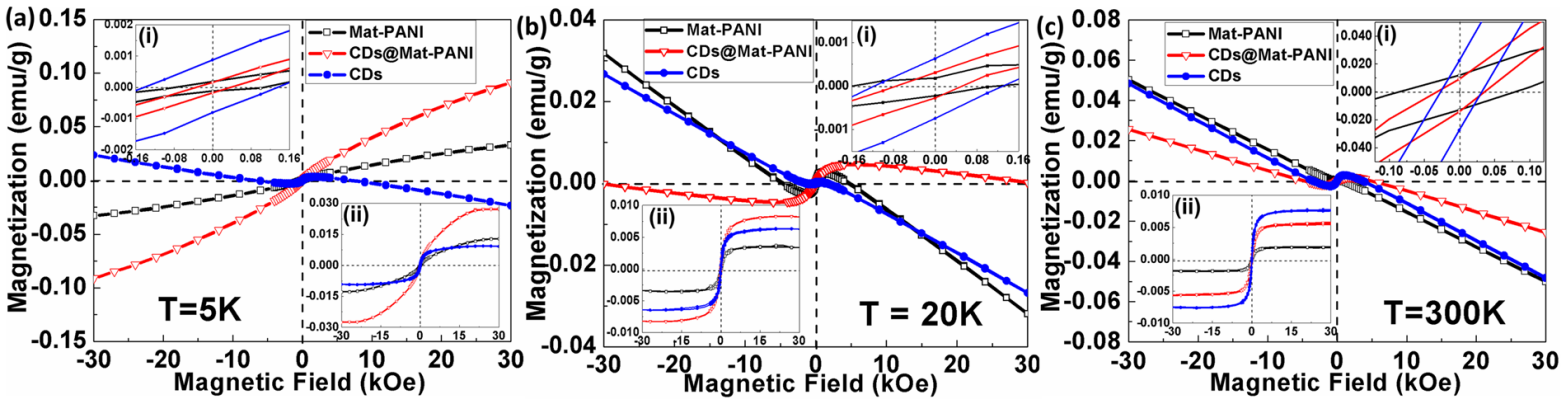
Magnetic hysteresis curves of the Mat-PANI, CDs@Mat-PANI and CDs at (**a**) 5 K, (**b**) 20 K, and (**c**) 300 K. The insets of (**a**(i)), (**b**(i)) and (**c**(i)) show magnetic hysteresis curves of Mat-PANI, CDs@Mat-PANI and CDs in the range of ±0.12 kOe. The insets of (**a**(ii)), (**b**(ii)) and (**c**(ii)) show the hysteresis curves after subtracting the diamagnetic component.

Figure 5 shows the magnetization data of the as-synthesized samples at 5, 20 and 300 K (± 30 kOe range), whereas the saturation magnetization (*M*_*S*_) and magnetic coercivity (*H*_*C*_) of Mat-PANI, CDs@Mat-PANI and CDs are collected in Table 1. Figure 5a(i), b(i) and c(i) show the hysteresis cycles in the ±0.12 kOe range, revealing magnetic ordering of samples CDs, Mat-PANI and CDs@Mat-PANI at 5, 20 and 300 K, respectively. Figure 5a(ii), b(ii) and c(ii) show the hysteresis cycles after removing the diamagnetic component. In addition to the novelty of RT magnetic ordering of CDs, the CDs@Mat-PANI sample shows enormous enhancement of *M*_*S*_ (about 200% (40%) at 5 (300) K) with respect to Mat-PANI (see data in Table 1). Neglecting any interaction between the two constituent phases (CDs and Mat-PANI), the expected *M*_*S*_ of the nanohybrid (CDs@Mat-PANI) at 5 K would be around 0.0116 emu/g (Vegard’s law), given the nominal CDs content of 0.315 wt%. However, the measured *M*_*S*_ of the nanohybrid is three-fold larger (0.0349 emu/g), revealing that there is indeed a strong interaction between CDs and Mat-PANI in the CDs@Mat-PANI.

**Table 1:** Saturation magnetization (*M*_*S*_) and magnetic coercivity (*H*_*C*_) of Mat-PANI, CDs@Mat-PANI and CDs at 300, 20 and 5 K.

T (K)	Mat-PANI		CDs@Mat-PANI		CDs	
	*M*_s_(emu/g)	*H*_c_ (kOe)	*M*_s_ (emu/g)	*H*_c_ (kOe)	*M*_s_ (emu/g)	*H*_c_ (kOe)
300	0.0055	0.04	0.0077	0.03	0.0019	0.09
20	0.0065	0.12	0.0085	0.07	0.0048	0.16
5	0.0116	0.08	0.0349	0.04	0.0079	0.14

Magnetic ordering in metal-free carbon nanomaterials has been attributed to the coupling of unpaired electrons^36, 37^ and supported by *ab initio* calculation^38–40^. Therefore, in CDs magnetic ordering is realized in the framework of coupling of unpaired electrons, likely associated with the shell layer, while ascribing to the CDs core region the quantum confined semiconductor nature. In contrast with GQDs, where ferromagnetism has been reported only at very low temperature^10, 11^ (2 K), an extra exchange interaction term, besides the usual spin-spin one (*J*_*ij*_ *S*_*i*_.*S*_*j*_), may account for RT magnetic ordering in CDs. The extra Hamiltonian term should describe a non-flat array of spins in CDs, as opposed to the flat array of spin centers in GQDs. On the other hand, ferromagnetism in PANI-related structures has been connected to the existence of two spin species associated with metallic islands (ordered crystalline region) immersed in low conducting matrix (amorphous regions)^41, 42^. The existence of crystalline and amorphous regions within PANI has been generally accepted and clear experimental evidences of it had been provided in the work of Beau *et al*.^43^. Furthermore, it has been shown that magnetic ordering in PANI-related materials increased while increasing the crystallinity degree of the polymer^44^.

Our findings regarding the enhancement of magnetic ordering in the nanohybrid sample fit nicely with the electron transfer model picture drawn to explain the observed PL redshift of the CDs while encapsulated into the Mat-PANI. As the electron density within the CDs increases the optical energy transition shrinks in due to shift of energy levels in the CDs. Worth mentioning that the CDs incorporated within PANI were slightly negatively charged, as assessed from zeta-potential measurements. Band-bending plus bandgap renormalization accounted for the reduction (−Δ*E*) of the effective bandgap of the CDs, namely −Δ*E* = *ρ* + 4.27*ρ* ^1/3^, where Δ*E* is expressed in meV and *ρ* (charge density) in units of 10^18^ cm^−3^ ^45, 46^. In the above equation for Δ*E* the linear term (*ρ*) describes band-bending whereas the non-linear term (4.27*ρ* ^1/3^) describes bandgap renormalization^45^. From the analysis of the *M*_*S*_ data at 300 K (5 K) of Mat-PANI, CDs@Mat-PANI and CDs, we found that in average 0.08 (0.81) electron was transferred from the Mat-PANI backbone (imine N-atoms) to each CD (see next paragraph). At 300 K this means an increase in electron density within each CD of about Δ*ρ* = 14.2×10^18^ cm^−3^. The estimated value of Δ*ρ* can be used in the above equation for −Δ*E* to obtain the energy shift of about 117 meV. On the other hand, at 300 K we found the redshift of the PL line equals to 110 meV (Fig. 3a). Deviation of only 7 meV from the calculated value to the experimental PL redshift is indeed an excellent result, given the difference between the two approaches, i.e. magnetization data used to extract Δ*ρ* versus optical data to assess PL shift (calculation has been shown in Supplementary information).

We can elaborate further the magnetic ordering enhancement in the nanohybrid as due to increase of density of unpaired electrons (spin-1/2 sites) within the Mat-PANI backbone via transferring electrons out from imine Nitrogen atoms into neighboring CDs, with consequent increase of electron density within the latter. The enhancement in *M*_*S*_ of the nanohybrid at 300 K (5 K) is about 0.0022 emu/g (0.0233 emu/g), meaning an increase of about 2.3723×10^17^ μ_B_/g (2.5124×10^18^ μ_B_/g). This implies that in average about 0.08 (0.81) electron is transferred to each CD at 300 K (5 K), thus increasing the number of unpaired electrons in the Mat-PANI backbone surrounding the CDs by the same amount. Given the spherical shape of the CDs with average diameter of about 2.21 nm (see Fig. 2a), the negative charge density transferred to each dot (Δ*ρ*) at 300 K amounts 14.2×10^18^ cm^−3^. Additionally, improved crystallinity in the CDs@Mat-PANI accounted for the increase of crystalline domain size from about 4.8 nm in Mat-PANI to 9.2 nm in CDs@Mat-PANI, with the corresponding reduction of tensile strain from about 6.2 to 1.8%, which supports the picture that the observed magnetic ordering enhancement is mainly due to electron density changes in the Mat-PANI triggered by strong interaction with CDs.

In summary, we reported room-temperature magnetic ordering in very small CDs and enhancement of ferromagnetism in CDs-incorporated mat-like PANI with a very low incorporation content of 0.315 wt%. The strong interaction between PANI and CDs occurred in a way of electron transfer from Mat-PANI imine N-atoms to the neighbouring CDs. The electron transfer process was confirmed by Raman, XRD and XPS data as well as PL redshift result, which increased electron density in the CDs while reduced the electron density in the PANI backbone. As a result, the increased number of unpaired electrons in the PANI backbone enhanced remarkably the *M*_S_ value of the nanohybrid (CDs@Mat-PANI).

## Methods

### Synthesis

In a typical synthesis process of CDs@Mat-PANI 2.23 g of aniline monomer (Shanghai Chemical Company) distilled under reduced pressure, 0.93 g of camphorsulfonic acid (CSA, Aladdin Industrial Corporation) and 10 mg of CDs (see panels (1), (2) and (4) in Fig. 1) were dispersed in 500 mL deionized water under magnetic stirring at 20–30 °C for 10 min. Next, the mixture was maintained at 30 °C in water bath for 1 h and then 500 mL aqueous solution containing 5.47 g of ammonium persulfate (APS, Sinopharm Chemical Reagent Company) was added to the above mixture in one portion (see panel (3) in Fig. 1). The resulting solution was stirred for a while to ensure complete mixing and then the reaction was allowed to proceed without agitation at 30 °C for 20 h. Finally, the yielded product was washed with deionized water and ethanol repeatedly until filtrate became colourless and then dried under vacuum at 80 °C for 24 h to obtain the dark-blue CDs@Mat-PANI sample. As a control, Mat-PANI sample was prepared by a similar *in-situ* polymerization procedure of the same amounts of aniline and CSA initiated by APS in deionized water.

### Characterization

Samples morphologies were observed by using scanning electron microscopy (Hitachi S-4800) and transmission electron microscopy (Hitachi H-800). The X-ray diffraction powder patterns were collected using a Rigaku D/MAX-cA X-ray diffractometer equipped with Cu Kα radiation (*λ* = 1.5406 Å) over the 2*θ* range of 5–60 degree. X-ray photoelectron spectroscopy studies were carried out on an ESCALAB250 spectrophotometer. Raman and photoluminescence spectra were obtained using a LabRAM HR800 Raman spectroscope and an UV532 spectrophotometer, respectively. Magnetic hysteresis curves were recorded with a SQUID magnetic measuring system (Quantum Design MPMS) in magnetic fields up to 30 kG at 5, 20, and 300 K.

## Electronic supplementary material

Supplementary information
