# Supplementary material for: Room-temperature Magnetism in Carbon Dots and Enhanced Ferromagnetism in Carbon Dots-Polyaniline Nanocomposite
Source: Sci Rep. 2017 May 19;7:2165. doi: 10.1038/s41598-017-01350-x (PMC5438339; doi:10.1038/s41598-017-01350-x)
Supplement: Supplementary file 1 — Supplementary information [file 41598_2017_1350_MOESM1_ESM.pdf]

Supplementary information

Room-temperature Magnetism in Carbon Dots and Enhanced Ferromagnetism in Carbon Dots-Polyaniline Nanocomposite

Jian Liu,[1,2] Hong Bi,[1,*] Paulo Cesar Morais,[1,3,*] Xiang Zhang,[1] Fapei Zhang,[4] and Lin Hu[4]

[1]College of Chemistry and Chemical Engineering, Anhui University, Hefei 230601, China
[2]Department of Chemical and Chemical Engineering, Hefei Normal University, Hefei 230601, China
[3]Universidade de Brasília, Instituto de Física, Brasília, DF 70910-900, Brazil
[4]High Magnetic Field Laboratory of the Chinese Academy Sciences, Hefei 230031, China
*These authors contributed equally to this work. Correspondence and requests for materials should be addressed to H.B. (email: bihong@ahu.edu.cn) or P.C.M. (email: pcmor@unb.br)

I. Supplementary data

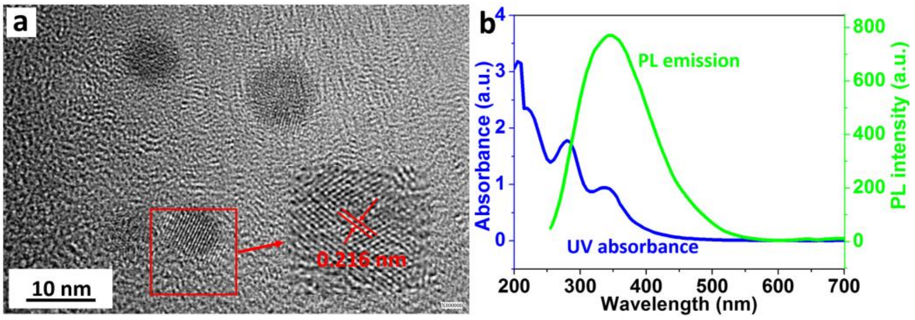

Figure S1. (a) TEM image of CDs and the inset is a HRTEM image of an individual carbon dot; (b) UV-Vis absorbance and PL emission spectra (λ_{em} = 310 nm) of negatively-charged CDs dispersed in ethanol.[S1,S2]

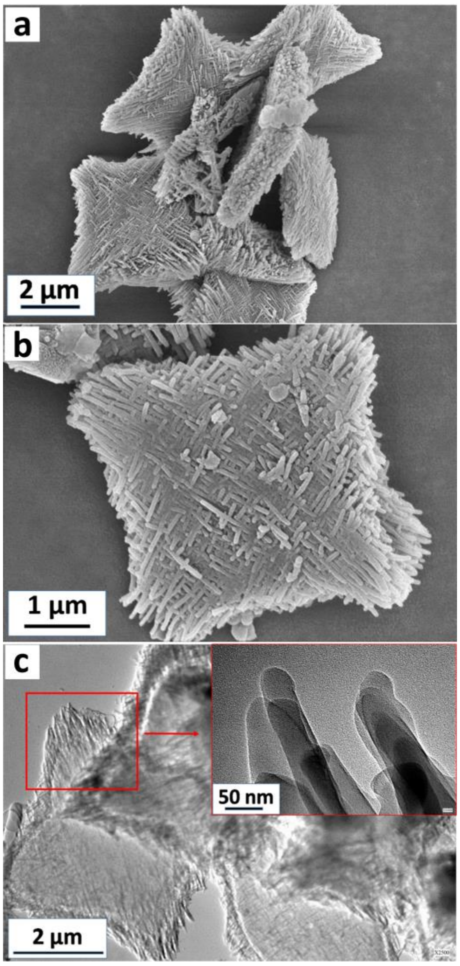

Figure S2. (a) Low-magnification and (b) high-magnification SEM images of Mat-PANI. c) HRTEM image of the Mat-PANI (the inset is a magnified image of the PANI nanofibers on the brim of a mat).

II. Calculation of Mat-PANI, CDs@Mat-PANI and CDs molecular weight (M_W)

Mat-PANI: $M_{w\text{Mat-PANI}} = (1/7)\text{CSA}+(6/7)\text{Aniline}$

$M_{w\text{Mat-PANI}} = (1/7)\times 234.3 + (6/7)\times 93.128 \approx 113.295$ g

CDs@Mat-PANI: $M_{w\text{CDs@Mat-PANI}} = 0.01\text{g CDs} + 3.16\text{g Mat-PANI}$

$M_{w\text{CDs@Mat-PANI}} = 0.00315\times 612.187+0.99685\times 113.295 \approx 114.866$ g

CDs: $m_{CD} = 2.16\ \text{g/cm}^3 \times (\pi/6)\ (2.21\times 10^{-7}\ \text{cm})^3 \approx 1.221\times 10^{-20}$ g

$M_{wCD} = 1.221\times 10^{-20}\ \text{g}/12.01115\times 1.66053\times 10^{-24} \approx 612.187$ g

$V = (\pi/6)(2.21\times 10^{-7}\ \text{cm})^3 \approx 5.65\times 10^{-21}\ \text{cm}^3$

*CDs from spherical-shaped (diameter-D) to cylindrical-shaped (height $2D/3$, diameter-D) with same volume: Cylinder lateral area = $(2/3)\pi D^2 \approx 10.2\ \text{nm}^2$ (then, 20 to 30 N-atoms around each CD)

Note: Units

mau = 1.66053×10^{-24} g

emu = $\times10^{-3}$ J/T

$\mu_B = 9.27402\times10^{-24}$ J/T

emu = 1.0783×10^{20} μ_B

ρ(graphite) = 2.09-2.23 g/cm^3 $\approx$ 2.16 g/cm^3

ξ(N: Mat-PANI) $\approx$ 2-3 N-atom/nm^2

Mat-PANI: 6 mol-PANI/1 mol-CSA

Table S1. Average molecular weights of Mat-PANI, CDs@Mat-PANI and CDs.

Sample	*Average molecular weight* (M_W)
Mat-PANI	113.295
CDs@Mat-PANI	114.866
CDs	612.187

III. Calculations

1. Magnetic calculation: Spin-centers (S-centers) and Nitrogen-centers (N-centers)

1.1. 5 K

(a) Mat-PANI: 0.0116 emu/g = $0.0079 \times 1.0783\times10^{20}$ μ_B = 1.2508×10^{18} μ_B/g

1 g (Mat-PANI) $\approx$ (1/113.295) mol = 0.0088265 mol

number of available N-centers: n(N) = $0.0088265\times6.023\times10^{23} \approx 53.162\times10^{20}$ N-center

fraction of active N-centers: f_o(N) = $(1.2508\times10^{18}/5316.2\times10^{18}) \approx 0.000235$ (0.0235%)

(b) CDs@Mat-PANI: $0.0349\times1.0783\times10^{20}$ μ_B = 3.7633×10^{18} μ_B/g

1 g (CDs@Mat-PANI) $\approx$ (1/114.866) mol = 0.008706 mol

number of available N-centers: n(N) = $0.008706\times6.023\times10^{23} \approx 52.435\times10^{20}$ N-center

fraction of active N-centers: f(N) = $(3.7633\times10^{18}/5243.5\times10^{18}) \approx 0.000718$ (0.0718%)

(c) CDs: 0.0079 emu/g = $0.0079\times1.0783\times10^{20}$ μ_B = 8.5186×10^{17} μ_B/g

1 g (CDs) $\approx$ (1/612.187) mol = 0.001633 mol

number of available S-centers: n(S) = $0.001633\times6.023\times10^{23} \approx 9.8385\times10^{20}$ S-center

fraction of active S-centers: f_o(S) = $(8.5186\times10^{17}/9838.5\times10^{17}) \approx 0.000866$ (0.0866%)

Therefore:

$(f/f_o) \approx (0.000718/0.000235) \approx 3.1$

and

M_S(CDs@Mat-PANI)/M_S(Mat-PANI) = (0.0349/0.0116) ≈ 3.0

1.2. 300 K

(a) Mat-PANI: 0.0055 emu/g = $0.0055\times1.0783\times10^{20}$ μ_B = 0.5931×10^{18} μ_B/g

1 g (Mat-PANI) $\approx$ (1/113.295) mol = 0.0088265 mol

number of available N-centers: n(N) = $0.0088265\times6.023\times10^{23} \approx 53.162\times10^{20}$ N-center

fraction of active N-centers: $f_o(\mathrm{N}) = (0.5931\times10^{18}/5316.2\times10^{18}) \approx 0.000112$ (0.0112%)

(b) CDs@Mat-PANI: $0.0077\times1.0783\times10^{20}\ \mu_B = 0.8303\times10^{18}\ \mu_B/g$

1 g (CDs@Mat-PANI) ≈ (1/114.866) mol = 0.008706 mol

number of available N-centers: $n(\mathrm{N}) = 0.008706\times6.023\times10^{23} \approx 52.435\times10^{20}$ N-center

fraction of active N-centers: $f(\mathrm{N}) = (0.8303\times10^{18}/5243.5\times10^{18}) \approx 0.000158$ (0.0158%)

(c) CDs: 0.0019 emu/g = $0.0019\times1.0783\times10^{20}\ \mu_B = 2.0488\times10^{17}\ \mu_B/g$

1 g (CDs) ≈ (1/612.187) mol = 0.001633 mol

number of available S-centers: $n(\mathrm{S}) = 0.001633\times6.023\times10^{23} \approx 9.8385\times10^{20}$ S-center

fraction of active S-centers: $f_o(\mathrm{S}) = (2.0488\times10^{17}/9838.5\times10^{17}) \approx 0.000208$ (0.0208%)

Therefore:

$(f/f_o) \approx (0.000158/0.000112) \approx 1.4$

and

$M_S(\mathrm{CDs@Mat\text{-}PANI})/M_S(\mathrm{Mat\text{-}PANI}) = (0.0077/0.0055) \approx 1.4$

2. Magnetic calculation: electron transfer from Mat-PANI to CDs

Number of CDs/g: $N^* = (0.00315\ \mathrm{g}/612.1873\ \mathrm{g})\times6.023\times10^{23} \approx 3.099\times10^{18}$ CDs/g

2.1. 5 K

Change of N-centers number: $\Delta N = (0.0349-0.0116)\times1.0783\times10^{20} \approx 2.5124\times10^{18}\ \mu_B/g$

Electron transferring to CDs: $\Delta N/N^* = (2.5124\times10^{18}/3.099\times10^{18}) \approx 0.81$ electron/CD

2.2. 300 K

Change of N-centers number: $\Delta N = (0.0077-0.0055)\times1.0783\times10^{20} \approx 2.3723\times10^{17}\ \mu_B/g$

Electron transferring to CDs: $\Delta N/N^* = (2.3723\times10^{17}/3.099\times10^{18}) \approx 0.08$ electron/CD

3. Optical calculation: electron density within CDs

3.1. 300 K

$\rho = 0.08/5.65\times10^{-21}\ \mathrm{cm}^3 \approx 14.159\times10^{18}\ \mathrm{cm}^{-3}$

4. Band bending (ρ) and band-gap renormalization ($K(\rho)^{1/3}$) in CDs (theory)[S3-S7]

4.1. 300 K

$-\Delta E = \rho + K(\rho)^{1/3}$; $K = 42.7\times10^{-6}$ meV×cm

$-\Delta E \approx 14 + 42.7(14)^{1/3} \approx 14 + 103 \approx 117$ meV

5. PL line shift (experimental)

331 nm to 341 nm (3.7432 eV to 3.6334 eV)

$\Delta E \approx 110$ meV

ΔE(deviation) ≈ 7/117 ≈ 6%

References:

S1. Wei, W. L., Xu, C., Wu, L., Wang, J. S., Ren, J. S. & Qu, X. G. Non-enzymatic-browning-reaction: a versatile route for production of nitrogen-doped carbon dots with tunable multicolor luminescent display. *Sci. Rep*. **4**, 3564 (2014).

S2. Chandra, S., Patra, P., Pathan, S. H., Roy, S., Mitra, S., Layek, A., Bhar, R., Pramanik, P. & Goswami, A. Luminescent S-doped carbon dots: an emergent architecture for multimodal applications. *J. Mater. Chem. B* **1**, 2375-2383 (2013).

S3. Chaves, A. S., Penna, A. F. S., Worlock, J. M., Weimann, G. & Schlapp, W. Optical control of two-dimensional electron density in a single asymmetric quantum well. *Surf. Sci*. **170**, 618-623 (1986).

S4. Cardoso, A. J. C., Morais, P. C. & Cox, H. M. Investigation of many-body effects in one-side modulation-doped InP-InGaAs heterostructure. *Appl. Phys. Lett*. **68**, 1105 (1996).

S5. Cardoso, A. J. C., Qu, F. & Morais, P. C. Optical control of the two-dimensional electron-gas density in single asymmetric quantum wells: Magnetic-field effect. *Phys. Rev. B* **60**, 4501 (1999).

S6. Aleksandrov, I. & Zhuravlev, K. Photoluminescence of GaN/AlN quantum dots at high excitation powers. *Phys. Status Solidi C* **7**, 2230-2232 (2010).

S7. Qu, F. & Morais, P. C. Investigation of the magnetic field dependence of electronic and optical properties in one-side modulation-doped GaAs/Ga1-xAlxAs quantum wells. *J. Quant. Electron* **34**, 1419-1425 (1998).
